# Time-dependency, predictors and impact on outcome of infarct transmurality assessed by magnetic resonance imaging in patients with st-elevation myocardial infarction reperfused by primary percutaneous intervention

**DOI:** 10.1186/1532-429X-13-S1-O1

**Published:** 2011-02-02

**Authors:** Suzanne de Waha, Steffen Desch, Ingo Eitel, Georg Fuernau, Philipp Lurz, Matthias Grothoff, Matthias Gutberlet, Gerhard Schuler, Holger Thiele

**Affiliations:** 1 University of Leipzig – Heart Center, Leipzig, Germany

## Introduction

Previous studies identifying predictors for transmural infarction in patients with ST-elevation myocardial infarction (STEMI) reperfused by primary percutaneous intervention (PCI), especially analyzing the time-dependency of transmural infarction, achieved inconsistent results and are limited due to small and highly-selected study samples. Furthermore it remains unclear whether transmural infarction assessed within the acute phase of STEMI is associated with adverse clinical outcome.

## Methods

STEMI patients reperfused by primary PCI (n=322) within 720 min after symptom-onset underwent contrast-enhanced magnetic resonance imaging (MRI) at a median of 3 days after the index event (interquartile range [IQR 2;4]). Patients were subcategorized into tertiles according to time-to-reperfusion: lower tertile (< 175 min), middle tertile (175-320 min) and upper tertile (> 320 min). Infarct transmurality was assessed by a score with late-enhancement grading as <25%, 25-50%, 51-75% and >75% transmurality analyzing all 17 left ventricular segments. Transmural infarction was assumed if the hyperenhancement extended 75% of wall thickness in at least one segment. Clinical follow-up was performed after a median of 20 months (IQR 13;29). The primary endpoint was defined as a composite of death and congestive heart failure.

## Results

Overall, transmural infarction occurred in 50.6% (n=157) of all patients. The infarct transmurality score progressed significantly with increasing ischemic time (2.7 [IQR 2.1;3.1] for <175 min, 3.0 [IQR 2.4;3.4] for 175-320 min and 3.2 [IQR 2.8;3.5] for >320 min; p<0.001).

Using multivariable logistic regression analysis including parameters such as post-PCI TIMI-flow, ST-segment resolution and maximum creatine kinase levels, time-to-reperfusion was identified as the only independent predictor for transmural infarction (odds ratio 1.02, 95%CI 1.01-1.03, p=0.03).

Furthermore, in Cox regression analysis neither the presence of transmural infarction nor the transmurality score were associated with the occurrence of the primary composite endpoint (presence of transmural infarction: hazard ratio [HR] 1.22, 95%CI 0.53-2.79, p=0.64 / transmurality score HR 1.07, 95%CI 0.73-1.58, p=0.74).

## Conclusion

In STEMI patients reperfused by primary PCI time-to-reperfusion is the only independent predictor for transmural infarction. However, infarct transmurality is not associated with the occurrence of death and congestive heart failure.

